# Supplementary material for: Comparative proteomics reveals a diagnostic signature for pulmonary head‐and‐neck cancer metastasis
Source: EMBO Mol Med. 2018 Aug 10;10(9):e8428. doi: 10.15252/emmm.201708428 (PMC6127892; doi:10.15252/emmm.201708428)
Supplement: Supplementary file 1 — Expanded View Figures PDF [file EMMM-10-e8428-s001.pdf]

Expanded View Figures

Figure EV1. Technical reproducibility.

A Pearson's correlation analysis of normalised SILAC ratios (log_2) of replicates (indicated as Rep 1 and 2) of all quantified proteins for representative SQCLC and HNSCC cases. Pearson's correlation coefficients (ρ) are indicated.

B Boxplot showing the numbers of quantified proteins of six squamous cell carcinoma tumour samples that were analysed on both an Orbitrap Fusion and a Q Exactive HF mass spectrometer (both Thermo Fisher). The central line in the boxes represents the median number of proteins over all samples, and upper and lower borders of boxes correspond to 25 and 75% quantiles. Whiskers indicate minimum and maximum.

C Technical reproducibility on different mass spectrometry platforms by direct comparison of the protein expression profiles of six squamous cell carcinoma tumour samples that were analysed on both an Orbitrap Fusion and a Q Exactive HF mass spectrometer (both Thermo Fisher).

 © 2018 The Authors

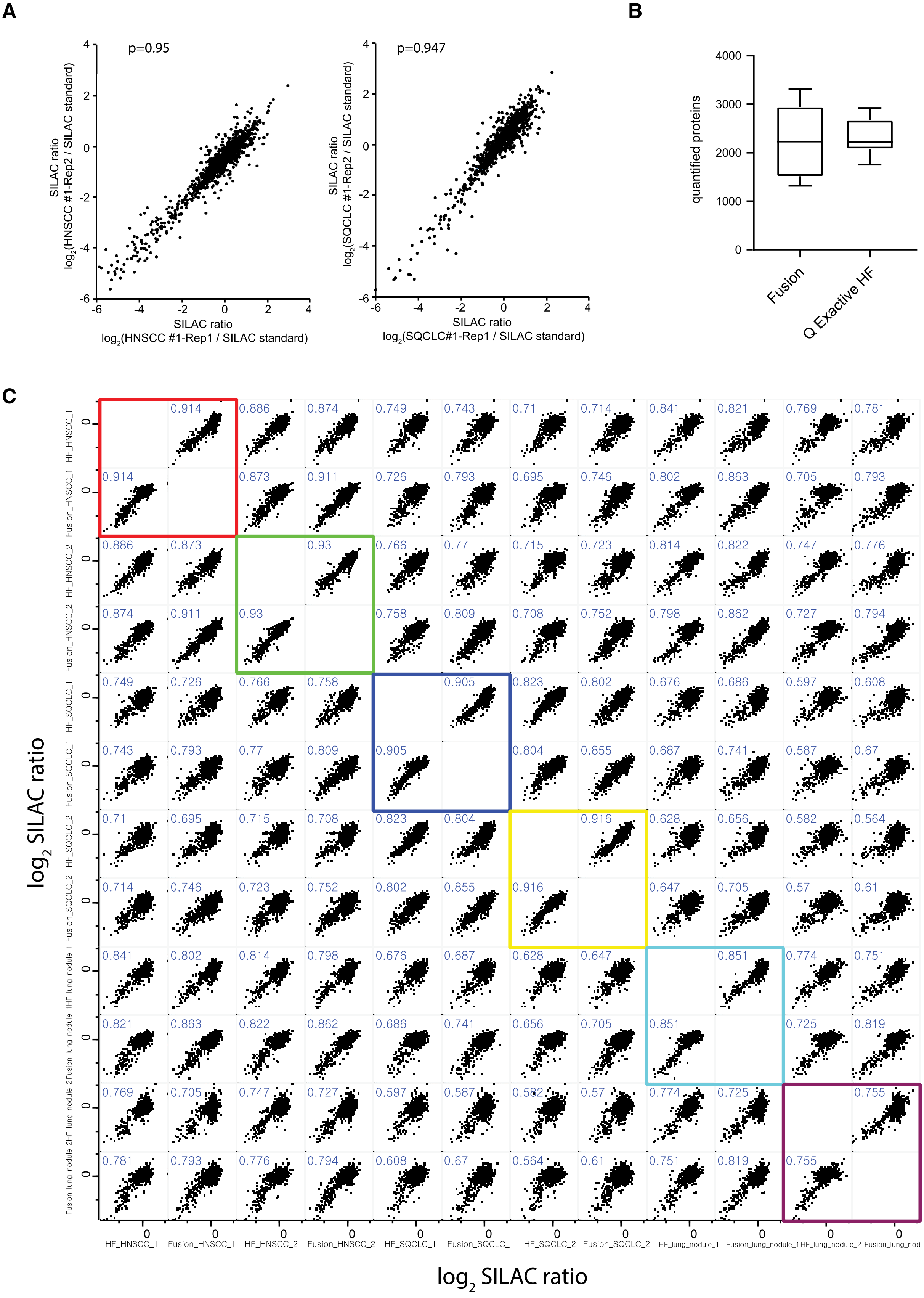
A
p=0.95
SILAC ratio
log2(HNSCC #1-Rep2 / SILAC standard)
SILAC ratio
log2(HNSCC #1-Rep1 / SILAC standard)
p=0.947
SILAC ratio
log2(SQCLC #1-Rep2 / SILAC standard)
SILAC ratio
log2(SQCLC#1-Rep1 / SILAC standard)
B
quantified proteins
Fusion
Q Exactive HF
C
log2 SILAC ratio
HF_HNSCC_1
Fusion_HNSCC_1
HF_HNSCC_2
Fusion_HNSCC_2
HF_SQCLC_1
Fusion_SQCLC_1
HF_SQCLC_2
Fusion_SQCLC_2
HF_lung_nodule_1
Fusion_lung_nodule_1
HF_lung_nodule_2
Fusion_lung_nod
log2 SILAC ratio

Figure EV1.

Figure EV2. Proteomic comparison of lung and head-and-neck carcinomas.

A Hierarchical clustering of the top 100 differentially expressed proteins between HNSCC (red) and SQCLC (blue).

B Immunohistochemical analysis of the expression of CK19 (P = 0.0001) in an independent cohort of 212 SQCLC and 343 HNSCC cases. Scale bar indicates 100 μm. Shown are mean values and standard deviation. Statistical significance was assessed using Wilcoxon–Mann–Whitney test.

C Immunohistochemical analysis of microvascular density (MVD) by CD34 staining of the SQCLC and HNSCC samples that were analysed by mass spectrometry. Scale bar indicates 50 μm. Shown are mean and standard deviation; statistical significance was assessed using Wilcoxon–Mann–Whitney test.

D Pearson's correlation analysis of mean normalised SILAC ratios (log_2) of quantified proteins related to pathways which were found to be most affected by mutations (PI3K, TP53 and NFE2L2/KEAP1 pathway) as defined by the KEGG database for all SQCLC and HNSCC cases. Pearson's correlation coefficients (ρ) are indicated.

© 2018 The Authors

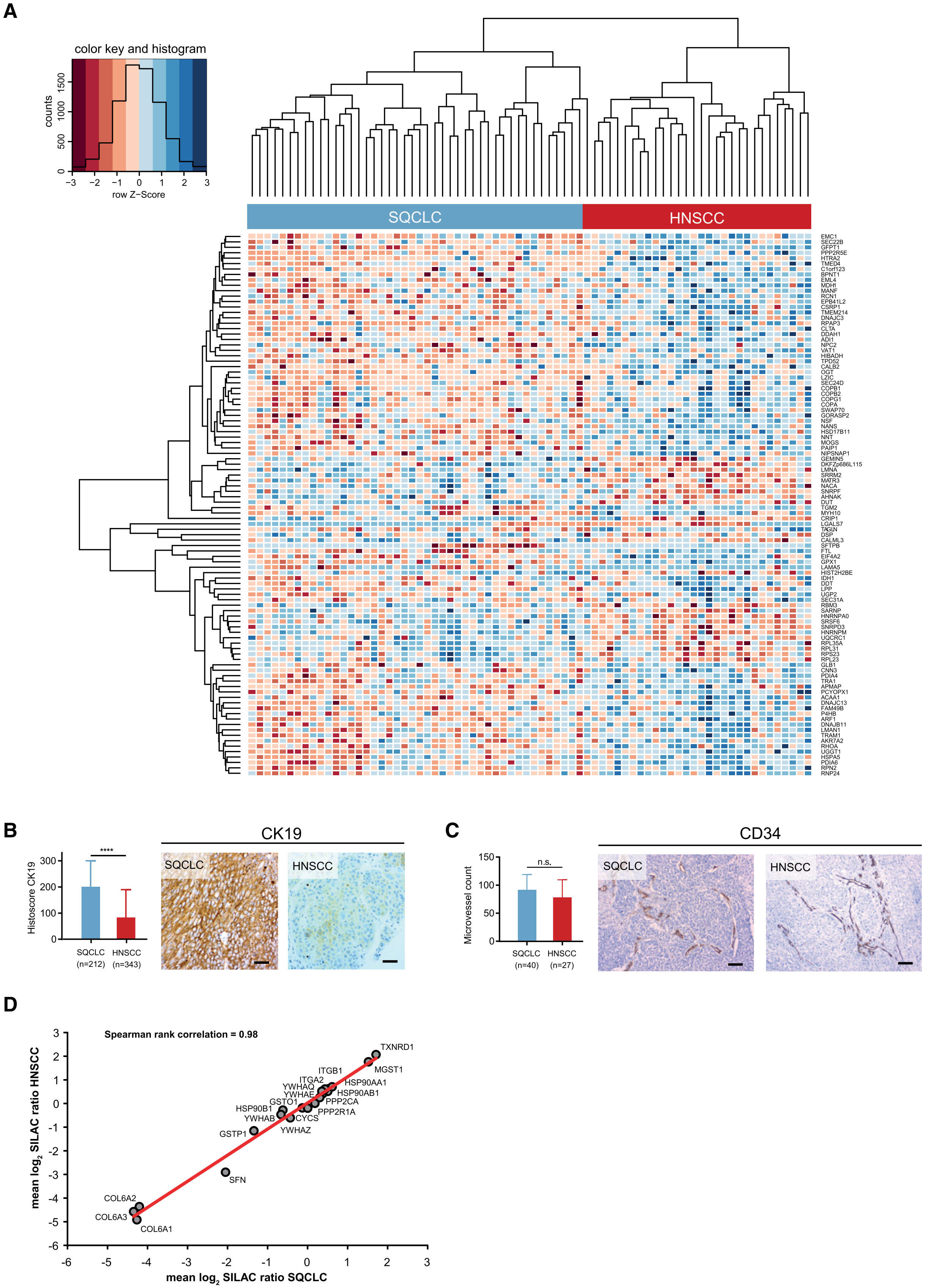
A
color key and histogram
counts
row Z−Score
SQCLC
HNSCC
B
CK19
Histoscore CK19
****
SQCLC (n=212)
HNSCC (n=343)
C
CD34
Microvessel count
n.s.
SQCLC (n=40)
HNSCC (n=27)
D
Spearman rank correlation = 0.98
mean log2 SILAC ratio HNSCC
mean log2 SILAC ratio SQCLC
TXNRD1
MGST1
ITGB1
ITGA2
HSP90AA1
YWHAQ
YWHAE
HSP90AB1
GSTO1
PPP2CA
HSP90B1
PPP2R1A
YWHAB
CYCS
YWHAZ
GSTP1
SFN
COL6A2
COL6A3
COL6A1

Figure EV2.

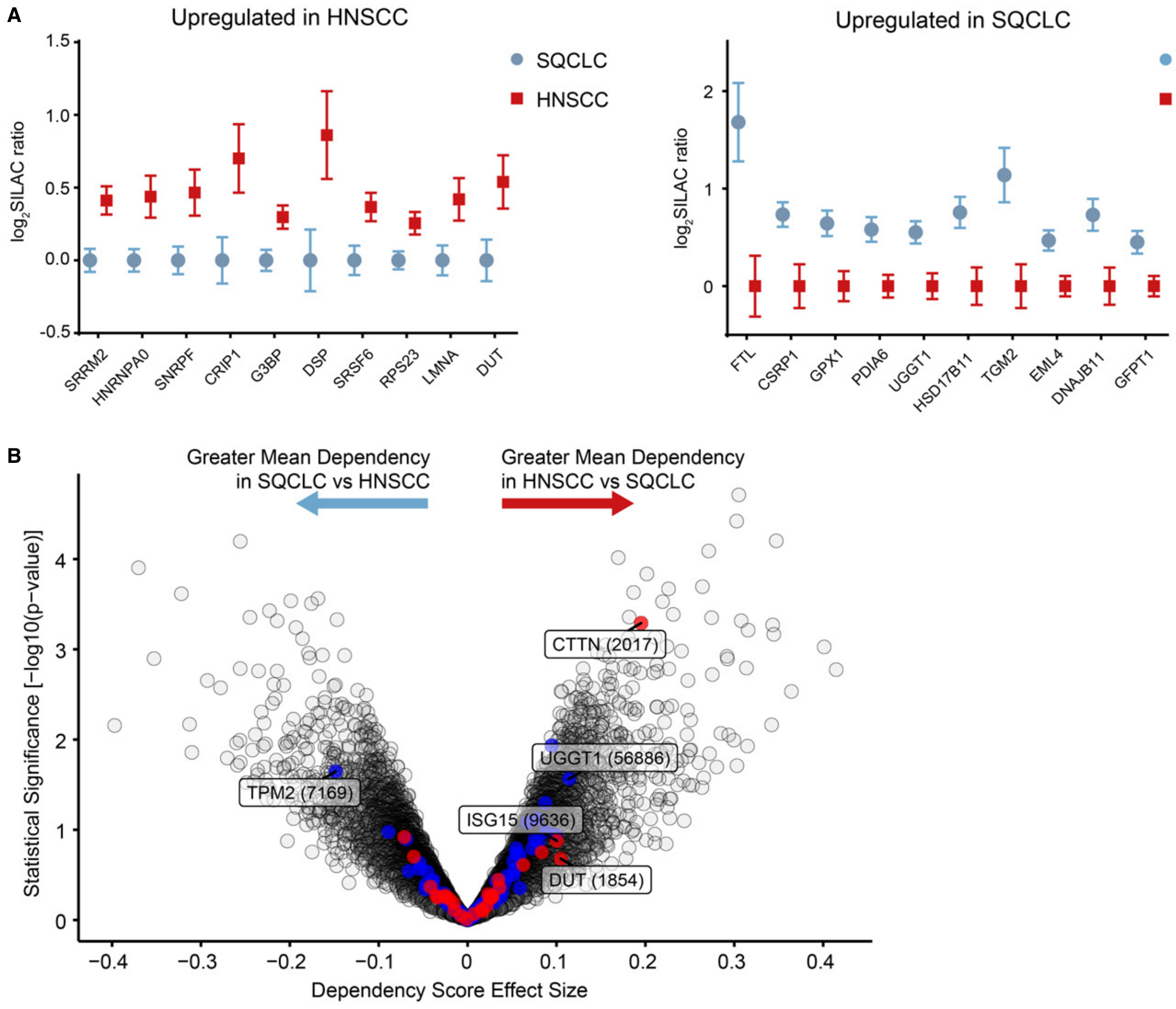

Figure EV3. Differentially expressed proteins in lung and head-and-neck carcinomas.

A Top 10 up-regulated proteins in HNSCC (top) and SQCLC. Whiskers indicate 95% confidence interval.

B Two-class comparison of genetic dependencies from a publically available genome-scale CRISPR-Cas9 screen of HNSCC (12 cell line) versus SQCLC (10 cell lines) identified a subset of differentially expressed proteins that were also differential dependencies in these tumour types. The *x*-axis represents the effect size of the mean difference of dependency scores in HNSCC compared to SQCLC cell lines. Positive effect size indicates a greater mean dependency in HNSCC; negative effect size indicates a greater mean dependency in SQCLC. The *y*-axis represents the statistical significance of differential enrichment calculated as −log10(*P*-value) from a two-sided *t*-test. The *P*-values used for this plot are uncorrected for multiple hypothesis testing. Highlighted in blue are the most differentially expressed proteins between HNSCC (red) and SQCLC (blue).

Figure EV4. Diagnostic signature for lung tumours of unknown origin.

A Heatmap showing somatic mutations in defined pathways and HPV status for 29 squamous cell lung tumours of unknown origin derived from HNSCC patients. (Blue, no mutation/no HPV detected; red, mutation/HPV detected; white, no data available).

B Representative histogram showing the SILAC ratio distribution between the Super-SILAC standard and a squamous cell lung tumour of unknown origin derived from an HNSCC patient.

C Classification results of the clinical score (left) and the SVM prediction model (right) for the 51 lung tumours of unknown origin. The p53 mutational status is shown. Red, HNSCC, blue, SQCLC.

© 2018 The Authors

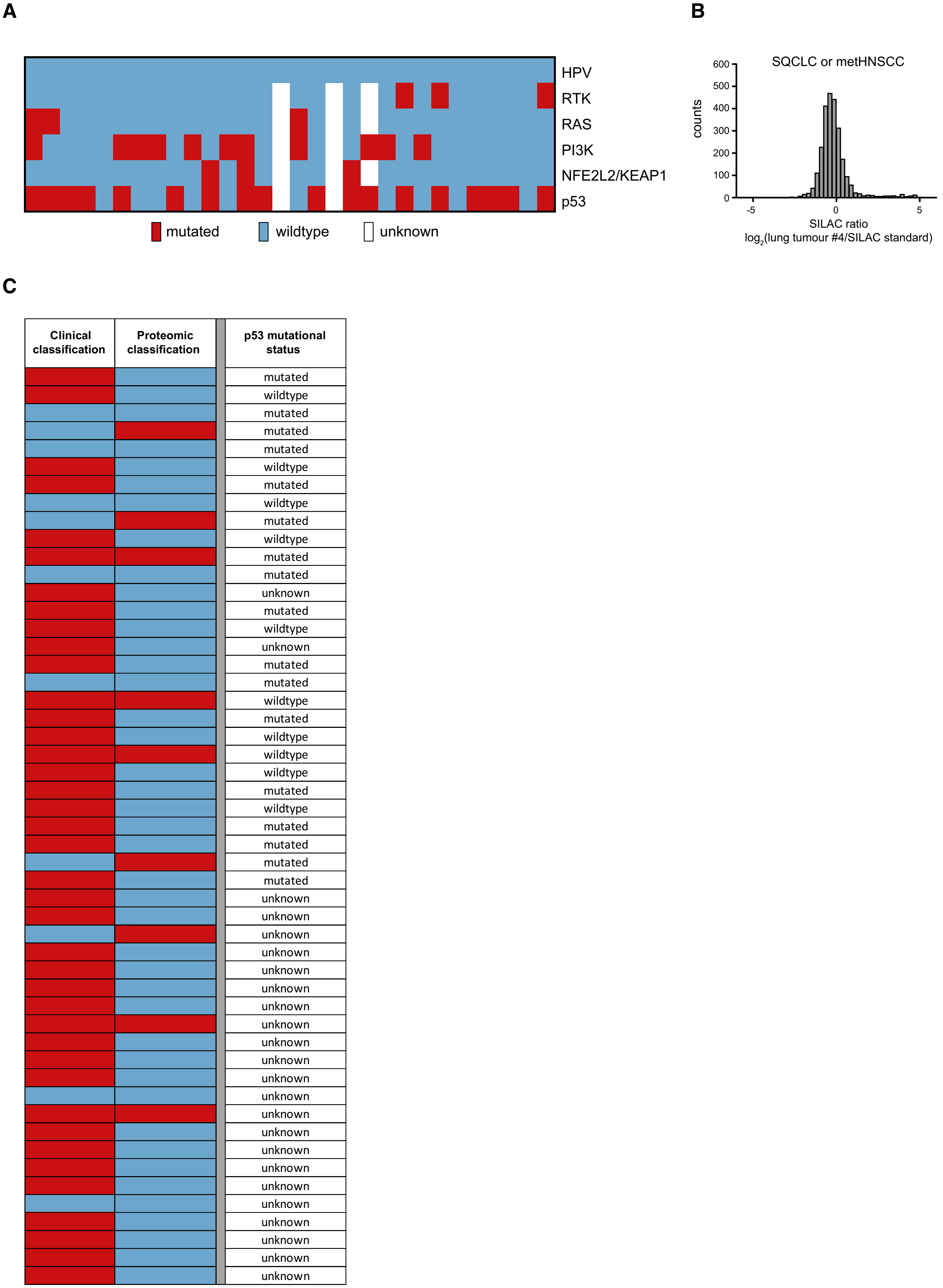
A
HPV
RTK
RAS
PI3K
NFE2L2/KEAP1
p53
mutated
wildtype
unknown
B
SQCLC or metHNSCC
counts
600
500
400
300
200
100
0
-5
0
5
SILAC ratio
log2(lung tumour #4/SILAC standard)
C
Clinical classification
Proteomic classification
p53 mutational status
mutated
wildtype
mutated
mutated
mutated
wildtype
mutated
wildtype
mutated
wildtype
mutated
mutated
unknown
mutated
wildtype
unknown
mutated
mutated
wildtype
mutated
wildtype
wildtype
wildtype
mutated
wildtype
mutated
mutated
mutated
mutated
unknown
unknown
unknown
unknown
unknown
unknown
unknown
unknown
unknown
unknown
unknown
unknown
unknown
unknown
unknown
unknown
unknown
unknown
unknown
unknown
unknown
unknown
unknown

Figure EV4.
